# Mortality Awareness: New Directions

**DOI:** 10.1177/00302228221100640

**Published:** 2022-05-07

**Authors:** Margot Kuylen, Shihui Han, Lasana Harris, Quentin Huys, Susana Monsó, Alexandra Pitman, Stephen M. Fleming, Anthony S. David

**Affiliations:** 1 Mental Health, Ethics and Law Research Group, Department of Psychological Medicine, 4616 King’s College London, London, UK; 2 Culture and Social Cognitive Neuroscience Lab, School of Psychological and Cognitive Sciences, 12465 Peking University, Beijing, China; 3 Department of Experimental Psychology, 4919 University College London, London, UK; 4 Division of Psychiatry and Max Planck UCL Centre for Computational Psychiatry and Ageing Research, 4919 University College London, London, UK; 5 Department of Logic, History, and Philosophy of Science, 16757 Universidad Nacional de Educación a Distancia (UNED), Madrid, Spain; 6 4919 UCL Division of Psychiatry, London, UK; 7 UCL Institute of Mental Health, 4919 University College London, London, UK

**Keywords:** death anxiety, cognitive neuroscience, suicide, terror management theory, functional magnetic resonance imaging

## Abstract

Thinking about our own death and its salience in relation to decision making has become a fruitful area of multidisciplinary research across the breadth of psychological science. By bringing together experts from philosophy, cognitive and affective neuroscience, clinical and computational psychiatry we have attempted to set out the current state of the art and point to areas of further enquiry. One stimulus for doing this is the need to engage with policy makers who are now having to consider guidelines on suicide and assisted suicide so that they may be aware of their own as well as the wider populations’ cognitive processes when confronted with the ultimate truth of mortality.

What happens when we think about our own death? How does this awareness affect our thinking and decision-making? These are interesting questions from a philosophical, psychological, neuroscientific and sociological perspective. But they may also have important implications for two highly emotive and contentious policy areas to which death is central: suicide and assisted dying. A central policy issue in these areas concerns when, if ever, a person has capacity to decide they want to die.

As a multidisciplinary group of mental health clinicians, philosophers, and social and cognitive neuroscientists interested in decision-making, we sought to explore mortality awareness in the light of recent research, to test whether it can inform the policy debate on such issues as suicide and assisted dying. Though research into mortality awareness is relatively new and questions remain as to how the gap between research and policy can be bridged: research will not directly answer our policy questions. But it may inform them – and, at the very least, policy decisions in these areas must be compatible with the science. This requires an effort from the sciences, most notably cognitive neuroscience, to expand beyond the narrow confines of its specialist interests into a broader area of discourse and consider things like the effects of depression and thinking about death on our capacity for decision making and future planning.

We held a workshop in June 2021 to explore new research into these and similar topics; below we attempt to distil some of the territory covered.

## The Concept of Death

Our starting point was recent conceptual work addressing the question of whether animals can understand death. Susana Monsó (2019) argues that a veritable concept of death would be found in a *cognitive,* rather than stereotypical, reaction to death: i.e. it would be (1) learned rather than innate, (2) flexible rather than rigid, (3) under cognitive control as opposed to automatic, (4) not tied to specific perceptual cues, (5) intra-individually and inter-individually heterogenous, and (6) not necessarily adaptive.

By extension, a concept of death is not binary – either present or absent – but rather exists on a spectrum: consider how children do not develop a concept of death overnight but attain it gradually. The “full concept” of death as we (adult humans) know it may not be attained by other animals, but this does not mean they cannot attain a concept of death to some degree. We should thus not ask whether animals have a full concept of death, but rather what a *minimal* concept of death would look like – and then see whether any animals may have a concept of death that meets this standard.

Monsó follows developmental psychologist Virginia Slaughter (2005) in considering that a full concept of death has seven components: (1) non-functionality, (2) irreversibility, (3) universality, (4) personal mortality, (5) inevitability, (6) causality, and (7) unpredictability. However, Monsó posits that only (1) and (2) are necessary to speak of a concept of death at all:

a creature can be credited with a minimal concept of death once she classifies some dead individuals as dead with some reliability, where “dead” is understood as a property that pertains to beings who: (a) are expected to have the cluster of functions characteristic of living beings, but (b) lack the cluster of functions of living beings, and (c) cannot recover the cluster of functions characteristic of living beings (Monsó, 2019, p. 9).

According to Monsó and Osuna-Mascaró (2021), the prerequisites for the development of a minimal concept of death are (1) a certain level of cognition, to process non-functionality and irreversibility, (2) a certain amount of experience with death, as the concept of death is not innate, and (3) some emotion (of any kind), to mediate the attention the animal must pay to the corpse.

Thus conceived, the concept of death is likely to be quite widespread in nature. Studies have shown that cognitive processing of non-functionality is common among animals: e.g. they develop expectations of how animate and inanimate object behave (Greggor et al., 2018) and attribute goal-directed behaviour only to biological entities (Kano & Call, 2014). Cognitive processing of irreversibility can simply be understood as the move from an expectation of functionality to an expectation of non-functionality, and is thus also common among animals – as are experiences of death and emotional incentives to pay attention to them.

But in nature, the concept of death probably emerges in more than its minimal form. In some cases, it may include some elements of causality and universality, as many animals are capable of associative learning and inductive generalisations. This means that while a minimal concept of death only enables an animal to acquire an explanation of what has happened to another animal who died, the natural concept of death also enables predictions: for example, they may gather that individuals who fall from trees can die of it. And this natural concept of death may, in turn, enable the animal to develop a concept of personal mortality: the notion that I can die. There exists some preliminary evidence that animals learn from dangers in their environment and adjust their behaviour accordingly, e.g., chimpanzees being very careful when crossing the road (Hockings et al., 2006), and crows recognising individuals who they perceive to be dangerous (Swift & Marzluff, 2015).

These arguments may contribute towards a more practical understanding of our clinical questions. For example, are there links between the way humans and animals react to death-related cues? And how can we disentangle stereotypical from cognitive responses to death?

## Suicide and Mortality Awareness

In the modern world, most of us may seem shielded from direct experiences of death and mortality. However, suicide and self-harm – pervasive throughout cultures and epochs – may intrude upon our consciousness. Apart from reports of suicide in the news and images in the arts and popular culture, more direct experiences of suicide touch many lives (Andriessen et al., 2017). Experiences of suicide bereavement are distressing for the individual and carry a risk of suicide (Pitman et al., 2014) – although studies on this issue are drawn specifically from high income countries in Scandinavia using registry data which do not include variables for religion or race, so questions on generalisability remain. Nevertheless, the experience of bereavement by suicide provides a powerful opportunity to learn about the effects of such confrontations with mortality on our personal psychology – including, from a clinician’s point of view, how it may influence decisions to end one’s own life.

Joiner’s Interpersonal Theory of Suicide (Van Orden et al., 2010) holds that, besides the feelings of being alone and feeling like a burden that engender suicidal ideation, the motivation to attempt suicide relies on acquiring the capability for suicide: a heightened degree of fearlessness and pain insensitivity that can override our evolutionary programming for self-preservation. Joiner and colleagues (Van Orden et al., 2010) propose that capability for suicide is not only caused by biological predisposing factors (such as genetic factors or low serotonin levels) but also by stress such as repeated exposure to painful and provocative life events such as physical abuse, the suicide of others, or “dry-runs” of practising or imagining one’s own suicide.

Systematic reviews show that risk of suicide is indeed increased in people bereaved by the suicide of, a child (Agerbo, 2005; Qin & Mortensen, 2003), parent (Garssen et al., 2011), sibling (Rostila et al., 2013; Tidemalm et al., 2011), cousin (Tidemalm et al., 2011), or partner (Agerbo, 2005; Erlangsen et al., 2017). To understand the underlying psychological processes in this context, Pitman and colleagues conducted a qualitative study with adults exposed to the suicide of a friend or relative (Pitman et al., 2017). Among other themes, thematic analysis showed that for some, suicide had become a more tangible option. A 19-year-old female, 9 years after her uncle’s suicide, spoke of “Knowing that if things get to be too difficult there’s a way out”, and a 25-year-old male, 9 years after his brother’s suicide, said that “If anything, it made it easier for me to consider and attempt suicide. If he had not done it, I probably would not have considered it in my lowest moments”. Similar qualitative insights were found in another UK study (Bell et al., 2015) and in a US study (Miklin et al., 2019).

This raises the idea of *suggestion* in relation to suicide: that exposure to another’s suicidal behaviour may increase the risk of self-harm, suicidal thoughts, suicide attempt, or suicide. A potential explanation is that exposure influences self-harm or suicidal behaviour through psychological processes like social modelling, emotional contagion, normalisation of suicidal behaviour, and cognitive availability (i.e. the awareness of suicide as an available option and knowledge of possible means of suicide, distinct from the physical availability of methods) (Florentine & Crane, 2010).

One implication is that population exposure to suicide should be examined more closely, for example by using cognitive measures for decision-making in distressed and non-distressed states, valid measures of fearlessness of death, and even neuroimaging – ideally with pre-exposure measures. There is also scope to address the cognitive availability of suicide through psychological interventions that tackle dysfunctional beliefs and fearlessness about suicide, and to reduce the rehearsal of suicide-related imagery by contextualising or altering it (‘imagery rescripting’). These findings also invite further questions, e.g. regarding the role of gender and culture in the acquisition of suicide capability, and how we can speak about suicide without worsening the problem through suggestion effects.

## Computational Approaches

Huys has used a computational approach to explore the cognitive computational mechanisms likely involved in decision-making in response to threats (see Huys et al., 2015). In his talk, he explored how death awareness may interact with four key components of decision-making: (1) threat responses, (2) opportunity costs, (3) temporal discounting, and (4) metacognition.

In the context of decision-making, death can be seen as the ultimate *threat*. In general, threat responses differ according to the *direction* and *distance* of the threat. The direction of a threat refers to whether we are approaching or rather escaping from it and influences our responses: anxiety allows an approach; fear facilitates escape (Blanchard & Blanchard, 1988; McNaughton & Gray, 2000; McNaughton & Corr, 2004). The distance of a threat refers to its intensity, and influences which neural systems are engaged: in both human and nonhuman animals, proximal threats engage the PAG (periaqueductal grey – which can generate species-specific threat and fear responses), whereas distal threats engage the PFC (prefrontal cortex) (Mobbs et al., 2007). Applying this to the human experience of death, the proximity of a death threat is likely to have a profound impact on the engagement of neural structures, as studied by Han and colleagues (2010; see point 4 below), and it is also likely to affect our reasoning abilities (although there may be substantial individual differences in how the distance of a threat is judged).

But death can also be seen as the ultimate *escape*. Millner et al. (2019) examined escape in people who are suicidal: participants could either press or not press a button to escape an unpleasant sound, or press or not press a button to avoid the sound altogether. Participants who were suicidal had an increased tendency to escape, but not to avoid (Anestis et al., 2014; Millner et al., 2019; Orbach, 2003) The association of suicidal ideation with an increased tendency to escape mirrors the qualitative reports above characterising suicide as a ‘way out’ when experiencing stress.

Apart from being a threat or escape, death can also be a reminder that life is short (*memento mori*). The focus does not here lie with death itself, but on the limited time we have to enjoy our lives – that is, in the language of computational decision-making, the limited time we have to obtain rewards. Studies suggest that foraging behaviour – looking for rewards in one’s environment – is driven by the average reward, which is the expected amount of reward per time unit. The higher the average award, the more vigour there will be in reward-seeking (Niv et al., 2007; Yoon et al., 2018). If we consider mortality awareness as a reminder of how little time we have to enjoy life’s rewards, then the average expected reward will increase in response to this reminder since the time unit in which rewards can be harvested is expected to decrease. This, then, may increase our reward-seeking vigour. Conversely, if you’re in a painful situation or suicidal, the opposite may happen, and reminders of how much time we have left may trigger an escape behaviour.

Another behavioural economics principle that can be brought in to illuminate mortality awareness is temporal discounting, which is the tendency to discount rewards that are further away in the future. Research suggests that depressed patients, suicide ideators, and those who engaged in low lethality suicide attempts are more impulsive (i.e. discount very strongly), whilst high lethality suicide attempters seem to discount less – and this relates to how well their suicide attempts were planned (Dombrovski et al., 2011).

Finally, studies show that humans strongly avoid an initial bad option, even if afterwards there may be better rewards down this path (Huys et al., 2012, 2015), and this process has been suggested to engage brain structures related to depression (Lally et al., 2017). This may be relevant to suicidal ideation if suicide is viewed as an escape and there is an inability to consider further suffering before achieving some resolution.

In conclusion, little research work has been done on the cognitive mechanisms underlying thoughts of death and suicidality, but this is an area of great potential interest. For example, it is possible to examine the cognitive choice processes at different stages of death confrontation – e.g., healthy versus terminally ill persons at different stages of illness – which is relevant to questions surrounding assisted dying. Further, it could be investigated which decision processes underlie a “valid” (i.e. capacitous) decision about one’s own death (i.e. when, if ever, suicide can be a rational decision).

## Functional Neuroanatomy

Discussions of cognitive and computational mechanisms, then, leads us to speculation regarding their neural correlates. Recently, a number of neuroimaging studies have been carried out that address specifically how the brain responds to thoughts of death, such as work by Han and colleagues using a variety of fMRI paradigms. In an initial study, participants were shown death-related words, negative words, and neutral words in 30-second intervals (Han et al., 2010). It was found that death-related words *decreased activity* in the mid cingulate and bilateral insulae (all parts of the salience network) relative to both neutral and negative words.

In a second study, Han and his colleague examined whether these mortality salience (MS) effects were *transient or sustained* by alternating death-related, negative, and neutral words as they were shown to participants. They found that the decreased activity in the salience network lasted, rather than rebounded immediately, after presentation of each death-related word (Shi & Han, 2013).

A third study tested whether thoughts of death also reduce subsequent *empathic* brain activity in the salience network (Luo et al., 2014). Participants were scanned while either undergoing MS priming or negative affect priming, followed by a calculation task to push mortality salience out of consciousness. They were then given images of people being subjected to painful and neutral stimuli. Findings showed that the group primed with MS showed reduced activity in the cingulate cortex in response to images showing others in pain, suggesting mortality salience reduces empathy.

In a fourth study, Han and colleagues studied the effects of mortality salience on functional brain activity involved in a learning task (Luo et al. (2019). Participants were again scanned during either MS or negative affect priming, shown a black screen for 7-8 minutes to induce a resting state, and then asked to perform a probabilistic learning task. It was found that MS decreased functional connectivity between the salience brain network and other networks during the resting state after thoughts of death. These differences could be predicted by self-reports of closeness to death, suggesting that thoughts of death indeed mediated the effect on the salience network. Another finding is that MS decreased cingulate and insular activity involved in subsequent reward learning, slowing down brain responses to reward and loss during the learning task. Finally, it was found that stronger functional connectivity between the mid-cingulate cortex and right ventral striatum predicted higher response accuracy in the neutral primed group, but not in the MS primed group, suggesting that mortality salience breaks the link between salience network activity and behaviour (Luo et al., 2019).

In sum, this body of work builds on behavioural studies of terror management theory (Greenberg et al., 1986) to suggest that thoughts of death, (1) reduce salience network activity and connectivity of its different nodes, (2) reduce the cingulate’s functional role of connecting other brain regions during the resting state, (3) decrease salience network activity in the following task regardless of task demands (e.g. empathy or reward learning), and (4) break the connection between salience network activity and behaviour.

This may explain the strong effect that thinking about death has on our behaviour: the cingulate cortex is involved in a range of processes such as time perception, decision-making, conflict monitoring, and emotion (Shackman et al., 2011). These studies show changes in mass univariate activity and it remains to be determined what type of information is being carried in the cingulate signal (for instance, about death-related content, or decision-making parameters, or both).

These findings invite many further questions: for example, might using different types of death-related words and sentences – e.g., those emphasising the shortness of life rather than the moment of death – produce different effects? Are mortality salience stimuli *coded* differently in the brain or are their effects due to affective responses? Does MS improve cognitive performance by subduing affective responses? And do MS effects differ depending on the age or cultural background of the subject (e.g., religious beliefs)? In theory, religion serves an anxiety buffering function where awareness of death is primed (Arrowood et al., 2018). However it goes without saying that not all religions are the same and those that do not emphasise concepts such as a just world – leaving this to the hereafter – may not serve this function (Bulut, 2021).

## Social Cognition

In order to integrate much of the foregoing, which has taken a conceptual or individualistic perspective, we must probe more deeply into what we might mean by ‘cultural background’ and indeed the social and political context of social behaviour. How might policy-makers, themselves human beings, be affected by mortality awareness when determining suicide and assisted dying policies? The necessary tools are found within social neuroscience. How we view and value each other – for example whether we view others as biological beings (similar to other plants and animals, making mortality salient and dehumanisation likely) or whether we view each other as human beings that reserve a special status relative to other forms of life with moral prohibitions against causing harm. Describing a person as a biological being draws focus to the person’s body rather than their mind, and thereby promotes MS as well as dehumanisation. A relevant process in this context is social cognition: our human ability to think about other minds and come to things like intentionality and culpability judgments, which is supported by a cortical network sometimes referred to as the Default Mode Network (due to its prevalence in resting state fMRI) or social cognition brain network (Mars et al., 2012).

An important feature of social cognition is its flexibility: humans have the ability to regulate the activity of this network – i.e., they can regulate whether they think about other minds (Harris, 2017). Disengaging this network may often lead to behaviour not typically reserved for other people, as others are ‘dehumanised’ by this process. Context, however, has a huge impact on whether and how we engage social cognition. Studies have found that, in certain contexts, social cognition brain network activity is reduced, e.g. when confronted with extreme outgroups (Harris & Fiske, 2006, 2007, 2011), when engaging in virtual violence (Mathiak & Weber, 2006), when confronted with sexualised images of women (Cikara et al., 2011), when buying or selling individuals in the labour market (Harris et al., 2014), or when retaliating against someone who used excessive punishment (Beyer et al., 2014).

But in other contexts, reduced social cognition may have the opposite effect – for example in legal decision-making. There is evidence that giving judges information about a defendant’s biological underpinnings influences the decisions they make about the person, mitigating the amount of punishment they are willing to assign to psychopaths (Aspinwall et al., 2012).

Harris and colleagues conceptually replicated this study, giving participants short vignettes that described people committing either very gruesome or not so gruesome crimes. Some vignettes included biological information about the person (e.g., ‘Robert has a genetic mutation that may be linked with higher aggressive rates in white males’), others included bold trait information (e.g., ‘Robert has an aggressive personality)’. Participants then made responsibility and punishment decisions within an MRI scanner—a social cognitive judgment and affective response respectively. It was found that people described by their traits were attributed more responsibility than people described by their biology, however, the harshness of punishment depended on how gruesome or disgusting the crime was, and not on the biology manipulation (Capestany & Harris, 2014), consistent with the psychological underpinnings of responsibility and punishment.

Since logical reasoning is theorised to underpin legal decision-making, the researchers then focused on the dorsolateral prefrontal cortex (DLPFC), a brain region identified during a logical reasoning localiser that participants completed before the main fMRI task. They observed that biological information reduced engagement of DLPFC for responsibility judgments, but only for the weakly disgusting cases; similarly, in punishment decisions, strong punishment information increased DLPFC compared to weak disgust cases, but only when biological information was given.

Thus, biological information impacts responsibility judgments when the crimes are not gruesome and facilitates the affective mechanism necessary to drive punishment. Focusing on people’s biology disrupts logical reasoning and social cognition. Within a legal context, emphasising people’s biology results in lower culpability and enables affect rather than logical to dictate punishment. Describing people as biological entities makes both dehumanisation likely and mortality salient, which has knock-on consequences for how legal decisions are made: mortality awareness can facilitate harsher punishments but reduce salience of mind leading to less responsibility for bad outcomes such as suicide.

## Discussion and Implications

At the end of the workshop, several future avenues for research were suggested. One important element to be examined is future thinking: thinking about oneself in the future and in different settings. For example, it is important to understand how this operates in people with life-limiting conditions and dementia who are thinking about their future in the context of advance care planning. A recent wide-ranging commission on ‘The Value of Death’ (Sallnow et al., 2022) explored many medically related aspects of death including the growth of palliative care and trends towards assisted dying, and recommended we all develop ‘death literacy’—the knowledge and skills that people need to navigate death systems. How this will affect our cognition remains to be studied. The question was also raised whether cognitive neuroscience methods can model future thinking around the actual confrontation with mortality in a way that allows us to interrogate it experimentally. Are there some existing paradigms to employ in this context, or could this be a new avenue of research? Can we use paradigms such as those described above, to characterise the affective cognitive processes involved at different stages of future thinking in ourselves and others, where death salience may play an evolving part? We invite further research into these and related questions so that when any policy decisions about suicide or assisted dying are made, they are informed by, and compatible with, the way our brains work.

## Note

1. Previous studies have shown that observing painful versus neutral stimuli activate cingulate and arterial insula cortices respectively.
